# Potential Ecological Risk and Human Health Risk Assessment of Heavy Metal Pollution in Industrial Affected Soils by Coal Mining and Metallurgy in Ostrava, Czech Republic

**DOI:** 10.3390/ijerph16224495

**Published:** 2019-11-14

**Authors:** Helena Doležalová Weissmannová, Silvie Mihočová, Petr Chovanec, Jiří Pavlovský

**Affiliations:** 1 Faculty of Chemistry, Brno University of Technology, Purkyňova 118, 612 00 Brno, Czech Republic; mihocova@fch.vutbr.cz (S.M.); chovanec1987@gmail.com (P.C.); 2 Department of Chemistry, Faculty of Materials Science and Technology, VŠB—Technical University of Ostrava, 17. listopadu 2172/15, 708 00 Ostrava-Poruba, Czech Republic; jiri.pavlovsky@vsb.cz

**Keywords:** heavy metals, soils, pollution assessment, ecological risk, human health risk

## Abstract

The heavy metal pollution of soils has become serious environmental problem, mainly in localities with high industrialization and rapid growth. The purpose of this detailed research was to determine the actual status of heavy metal pollution of soils and an assessment of heavy metal pollution in a highly industrialized city, Ostrava, with a history of long-term impacts from the metallurgy industry and mining. The ecological risks to the area was subsequently also assessed. The heavy metals Cd, Hg, Cu, Mn, Pb, V, Zn, Cr and Fe were determined in top-soils (0–20 cm) using atomic absorption spectrometry (F AAS, GF AAS) from three areas with different anthropogenic loads. The obtained data expressed as mean metal concentrations were very varied among the sampled soils and values of all analyzed metal concentrations were higher than its background levels. To identify the ecological risk and assessment of soil pollution, various pollution indices were calculated, such as single pollution indices (I_geo_, CF, EF, PI) and total complex indices (IPI, PLI, PI_Nemerow_, C_deg_, mC_deg_, E_r_ and PERI). The identification of pollution sources was assessed using Pearson’s correlation analysis and multivariate methods (HCA, PCA/FA). The obtained results confirmed three major groups of metals (Fe–Cr, Pb–Cu and Mn–V). A human health risk was identified in the case of Pb, Cd and Cr, and the HI value of V for children also exceeded 1.

## 1. Introduction

Metal-polluted soils have become a global and major environmental problem in many parts of the world. Many research studies are focused on monitoring heavy metal concentrations in soils, such as Cu, Zn, Fe, Mn, Co, Ni, Pb, Cd, Cr, As and Hg, which very often have been extensively studied, mainly in urban, agricultural and industrial affected soils [1,2,3,4,5,6]. The pollution of soils together with water and wind erosion, soil degradation, loss of organic matter, disruption of water cycles and acidification processes have adversely impacted soil functions. Heavy metals originating from anthropogenic activities are among the most common and oldest environmental contaminants. The occurrence of heavy metals and some semi-metals in the environment (soil, water and air) increases the potential intake of these usually toxic substances by the living organisms but also accumulate in various body organs (kidney, liver and bone) and cause damage to body systems (nervous, skeletal, endocrine and immune, circulatory, etc.) [7,8,9]. The high soil pollution by heavy metals results in enormous potential ecological risks in threatened areas with typical rapid urbanization, industrialization and agricultural development. These global pollution trends state serious problems to human health and disrupt the balance of local ecosystems. Heavy metal pollution is hidden, permanent and irreversible due to the basic character of heavy metals, such as accumulation, persistence and low mobility in soils. Growing industrial development, waste production and agricultural emissions of heavy metals have major roles in the rapid increase of metal pollution in soils; anthropogenic activity is therefore the reason for the rapidly growing pollution trend [10,11,12,13]. Mining and metallurgical activities also affect the presence of other elements in soil, in particular As, Cd and Zn. High levels of metals and pronounced soil contamination can be detectable at a distance nearly 1.5 km from the source [4,6,14,15,16,17,18]. Intensive mining industry caused severe pollution in some areas in the past decades in the Czech Republic, mainly Ostrava. During long periods, an enormous amount of mining, processing and metallurgical waste were produced. General problems in these areas were slow, insufficient or missing remediation processes. Currently, the Ostrava region is the only locality with mining of black coal in Czech Republic. During the period 1990–2001, numerous coal mines were closed and only three coal mines are still operated. This fact led to a change in the usage of high-quality black coal and its substitution with cheaper and lower quality brown coal, mainly used in many household heating systems in the Ostrava region.

The main objectives of this study are (1) determination of total concentrations of Cd, Hg, Cu, Mn, Pb, V, Zn, Cr and Fe and pollution degrees in soils in relation to industrial activities in Ostrava; (2) application and calculation of single and total complex indices for pollution assessment of soils and assessment of environmental risk in this area; (3) identification of pollution sources of heavy metals in soils from Ostrava based on multivariate statistical method; (4) identification of ecological risk in Ostrava; and (5) evaluation of carcinogenic and non-carcinogenic risk in Ostrava, as well as health risk assessment.

## 2. Materials and Methods

### 2.1. Study Area and Sample Collection

The Ostrava region is located in the Moravia–Silesian Region in north-eastern Czech Republic, near the border with Poland, and lies in the center of the Ostrava Basin that is a geomorphological unit in North Moravia and Silesia. This region is one of the most industrialized and urbanized sites in Central Europe and the characteristic long-term problems in this region are enormous air pollution due to anthropogenic activity and many pollution sources, which are some of the highest in Europe. The Ostrava agglomeration is densely populated (978/km^2^) and with area 331.53 km^2^. The rapid economic and urbanization trend were in the last century due to fast development of coal production, steel and heavy industries (steelworks, metallurgical industry, production of steel alloys, metalworking, etc.). A high-concentration industry, large density of local coal combustion due to local heating needs and a very high infrastructure for mainly transport, all belong among the highest pollution sources in this region. Many localities in this area are therefore seriously and long-term affected. Pollution of air, soil and water (emissions) is the most serious problem in Ostrava.

**Figure 1:**
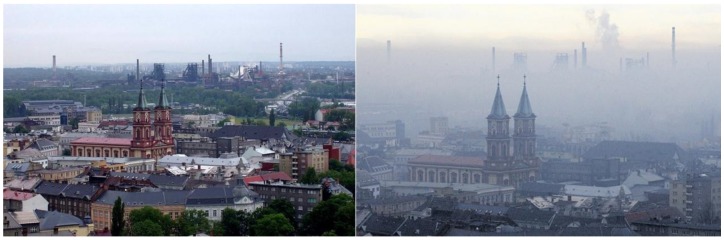
A typical smog situation in Ostrava—view of the center of Ostrava and Vítkovice (the metallurgy and steel machinery group) in various smog situations.

The main part of Ostrava is characterized by industry with high emissions and typical there is enormous metallurgical production that is not similar in the Czech Republic. The quality of soil, air water, indeed the whole environment, is strongly affected by high dense transport, local coal combustion, non-maintained areas and the effects of mining and other anthropogenic activities. The high and usually occurring smog situation is typical for the Ostrava agglomeration (Figure 1) and the amount of particular matter (PM_10_), hydrocarbons (mainly benzo(a)pyrene), heavy metals (Pb, Cd, Cr, Hg, etc.), as well as oxides of sulfur and nitrogen are often exceeded. Additionally, several stationary industrial sources of pollutants (e.g., metals, particulate matter, nitrogen oxides, sulfur dioxide, and benzo(a)pyrene) exist directly in the city or in the surroundings.

**Figure 2:**
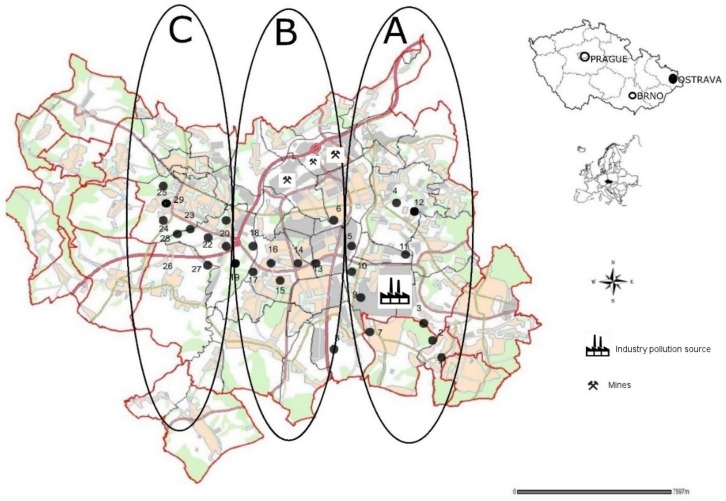
Location of the study area and sampling site in Ostrava (created from MAP 2019 [23]), modified and added onto the sampling points and sites **A**, **B** and **C**.

Parallel sampling series were carried out at 29 sites and from a depth of 20 cm depending on the distance from the major pollution source and soil samples were collected in accordance with ISO 11465 (1993), ISO 10381-5 (2005) and ISO 10390 (2005). All sampling sites were identified by a GPS system (TOMTOM) and sites are shown in Figure 2. Soil samples were transferred in polyethylene zipper bags preventing sample oxidation to the laboratory for chemical analysis [19,20,21,22].

The soil samples were classified into 3 groups depending on the pollution source distance (A: 0–5 km, B: 5–10 km and C: 10–16 km) and type of land utility. The samples from Site A were characterized as industry affected soils; Site B as urban land; and Site C as suburban with high traffic loads.

### 2.2. Chemical Analysis

The samples were air-dried at laboratory temperature (22 ± 2 °C) until constant weight and afterwards sieved through a 2 mm sieve, firstly, and again sieved through a 150 µm nylon sieve. The dry matter of soil samples (1 g) was determined by the gravimetric method (ISO 11465-1993). Measurements of pH were determined electrochemically using the WTW pH 320 system in accordance of method ISO 10390-2005. The air-dried soil samples (0.5000 ± 0.001 g) were digested with concentrated HNO_3_ (2 mol L^−1^, p.a. purity, Merck & Co., Inc.) and continuously shaken (16 h, at 22 ± 1 °C). Soil acid suspensions were filtered through a 0.45 µm membrane filter. The glassware were cleaned in HNO_3_ (10%) by soaking for at least 24 h and washed in deionized water. Milli-Q water (resistivity over 18 MΩ/cm) was used for preparing soil extraction solutions, as well as standards and blank solutions. The total concentration of Cd and V were determined by graphite furnace atomic absorption spectrometry (GF AAS; ZEEnit model AAS 60—Analytical Jena). The total concentrations of Pb, Zn, Cu and Mn were determined by flame atomic absorption spectrometry (FAAS; SpectrAA 30, Varian). The residual mercury content in the solid soil samples and extraction solutions were determined by a mercury analyzer AMA 254 (Advanced Mercury Analyzer).

The certified reference materials (QCM) METRANAL 34 and certified standard solution of CRM ASTASOL were used for QA/QC. The quality control checks were performed using reagent blanks, triplicate samples and certified quality control materials (QCM) METRANAL 34. The quality control materials METRANAL 34 was used to verify the accuracy of the analytical procedures The check standard of mercury was prepared by mixing 0.5 mL concentrated HNO_3_ and 0.5 mL concentrated HCl and 0.5 mL K_2_Cr_2_O_7_ (1%) solution and adding 10 µL of the standard mercury solution (ANS024, 1 g L^−1^) in 50 mL. The quality control of soil analysis was performed using a certified standard solution of CRM ASTASOL—Cd (ANS010), V (ANS065), Pb(ANS041), Zn (ANS069), Cu (ANS015), Mn (ANS033), Cr (ANS033), Hg (ANS024) from ANALYTIKA^®^, spol. s r.o., CZ. The matrix composition of METRANAL 34 is presented in Table S1 (Supplementary Materials). Each sample was analyzed in triplicate and RSD (relative standard deviation) varied from 3% to 10%. The standard errors for the measurements in the soil samples were in the range 0.1–0.5%. The limit of detection (LOD) and limit of quantification (LOQ) were also determined LOD—Pb 0.95, Cd 4.09.10^−4^, Cu 0.25, Cr 0.09, Fe 2.51, Mn 0.20, V 9.15.10-3, Zn 0.26 and LOQ Pb 3.17, Cd 1.37.10-3, Cu 0.95, Cr 1.08, Fe 3.26, Mn 0.67, V 3.05.10-2 and Zn 0.87 mg L^−1^. The experimentally obtained data were calculated on the dry weight and were expressed as means.

### 2.3. Pollution and Ecological Risk Assessment

Single and total complex indices were used for assessment of soil pollution by heavy metals in Ostrava (A—industrialized sites; B—sites with high urban density and traffic; and C—the peripheral part of Ostrava). The calculation methods (equations) of single and total complex indices used in this study are summarized, calculated and mentioned in many scientific articles, almost over several hundred articles, which are presented in (Table S2, Supplementary Materials) [6,24,25,26]. The pollution indices present the most effective tools of heavy metal pollution assessment in soils in comparison to total metal concentration in soils. The single indices calculated in this study allowed assessing the soil pollution with individual heavy metals and also calculated for each metal on the base their total concentration in soils and its contents, geochemical background or preindustrial levels. The geoaccumulation index (I_geo_) allows assessment of heavy soil metal contamination on the base metal concentrations in the soil sample (topsoils) and the geochemical background of the metal as the reference level. The enrichment factor (EF) allows assessment of the degree of intensity of anthropogenic activities; the reference background concentrations of Fe, Al, Ca, Ti, Sc or Mn are often used for calculation and identify anthropogenic impact of heavy metal concentrations in the soil. The contamination factor (CF) is used for assessment of soil pollution in relation to heavy metal concentration in soils and pre-industrial reference levels, and allows to identify the heavy metal pollution impact of soil due to anthropogenic activities [27,28]. The total complex indices presented in this study include the integrated pollution index (IPI), integrated threshold pollution index (IPI_T_), pollution load index (PLI), degree of contamination (C_deg_), modified contamination factor (mC_deg_) and Nemerow pollution index (PI_Nemerow_), and these indices are calculated on the basis of single indices and serve to assess the degree of contamination in a soil environment [29,30].

The integrated pollution index (IPI) is expressed as the mean value of single pollution indices of the metal (PI) that is calculated as the ratio mean of metal concentration in the soil and the geochemical background of individual metals. The integrated threshold pollution index (IPI_T_) is also called multielement contamination and can identify the soil pollution with regard to tolerable limits (national guidance permissible limits) of individual metals and also assess the common effect of metals in soil [31]. The pollution load index (PLI) serve to identify the accumulation levels of heavy metal contamination in soils and is calculated on the basis of the contamination factors (CF) of individual metals. The PLI provides a simple and comparative mean for assessment of quality in a site [32,33]. The degree of contamination (C_deg_) and modified contamination factor (mC_deg_) are used for assessment of metal pollution status or level in soils. The Nemerow Pollution Index (PI_Nemerow_) evaluates the total soil pollution degree with respect to contents of all determined heavy metals and their geochemical background and assesses the quality of soils. Ecological risk assessment is expressed as the potential ecological risk index (PERI, or RI), and combine the single index of ecological risk factor ($E_{r}^{i}$) and the toxic response factor of individual metal $T_{r}^{i}$, whereas PERI (RI) is expressed as the sum of potential risks of individual metals $E_{r}^{i}$ that is calculated multiplying the toxic-response factor of metal $T_{r}^{i}$ by contamination factor (CF). The toxic response factor $T_{r}^{i}$ is defined as 40, 30, 10, 5, 5, 5, 2, 2, 1, 1 and 1 for Hg, Cd, As, Cu, Pb Ni, Cr, V, Zn, Mn and Ti. [27,34,35,36,37,38,39,40,41,42].

### 2.4. Health Risk Assessment

Health risk assessments of heavy metals in soils are widely used to allow quantification of carcinogenic and non-carcinogenic risks to humans via ingestion, inhalation, dermal and dietary exposure pathways. The basic equation for human exposure and evaluation of health risk assessment, including the carcinogenic and non-carcinogenic risks of heavy metals, are the based on the recommendations and methodology of United States Environmental Protection Agency (1989) (USEPA 2001) [43,44,45,46,47,48].

The chronic daily intake (*CDI*; mg/kg/day) of heavy metals via ingestion (*CDI_Ing_*), dermal contact (*CDI_Derm_*) and inhalation (*CDI_Inh_*) for children and adults were calculated by formulas

(1) $$CDI_{Ing}=\;\frac{C_{soil\;}\;.\;\;R_{Ing}\;.\;\;EF\;.\;\;ED}{BW\;.\;\;AT}.\;10^{-6},$$

(2) $$CDI_{Derm}=\;\frac{C_{soil}\;.\;SA\;.\;AF\;.\;ABS\;.\;EF\;.\;ED}{BW\;.\;AT}.\;10^{-6},$$

(3) $$CDI_{Inh}=\;\frac{C_{soil}\;.\;EF\;.\;ET\;.\;ED}{PEF\;.\;BW\;.\;AT},$$

where *C_soi_*_l_ is the concentration in soil (mg/kg), *R* is the rate of ingestion (100 mg/day (adult), 200 mg/day (children)), *EF* is the exposure frequency (350 d/a), *ED* is the exposure duration (24 years (adult), 6 years (children)), *ET* is the exposure duration (24 h/d), *BW* is the body weight of the exposed individual (70 kg (adult), 15 kg (children)), *AT* is the averaging time (days), 365 × *ED* adult/children, *SA* is the exposed skin area (5700 cm^2^); *AF*—adherence factor: 0.07 mg·cm^−2^, *ABS* is the dermal absorption fraction: 0.03 (As), 0.001 (other metals), and *PEF* is the particle emission factor: 1.36 × 10^9^ m^3^ kg^−1^ [49,50,51,52].

The hazard index (*HI*) indicates the cumulative non-carcinogenic risk. The *HI* is equal to the sum of the hazard quotient (*HQ*) that express non-carcinogenic risk from individual heavy metals, and *HQ* is calculated according the equations

(4) $$HQ=\;\frac{CDI}{RfD},$$

(5) HI= ∑HQ,

where *RfD* is the chronic reference dose of the toxicant (mg kg^−1^ d^−1^) and are different for each element of heavy metal (Cu—0.0371, Co—0.02, Fe—0.7, Pb—0.0035, Zn—0.3, Cr—0.003, Cd—0.001, Ni—0.0008 and As—0.0003). The metals such as As, Cd, Cr and Pb are classified into metals with carcinogenic risk, and Fe, Zn, Cu, Ni and Co are non-carcinogenic.

If the *HI* is <1, no risks from non-carcinogenic effects probably occurred, and if the *HI* is >1, adverse health effects are possible, and the probability of effects increases with the increases in the *HI* values. The value of carcinogenic risk (*CR*) can be expressed as

(6) $$CR=\;CDI_{x}\;.\;SF,$$

(7) LCR= ∑CR ,

where *SF* is the carcinogenicity slope factor, and values for Cd, Cr, Pb and As are 6.3, 0.5, 0.0085 and 1.5 mg/kg/day. If *CR* < 10^−6^ the carcinogenic risks to human health from the soil can be considered as negligible, the range 1. 10^−6^–1.10^−4^ could be considered as posing an acceptable risk to humans and if *CR* > 10^−4^ presents a high risk for the development of cancer in humans. The sum of *CR* represents total cancer risk over lifetime *LCR*. The acceptable threshold value of CR is 1.0.10^−4^ and the range of tolerable value *LCR* is from 1.0.10^−6^ to 1.0.10^−4^ [49,50,51,52,53,54].

### 2.5. Data Analysis

The statistical analysis was carried out using descriptive statistics and multivariate statistical techniques—hierarchical cluster analysis (HCA) and principal component analysis (PCA) with factor analysis (FA). The multivariate statistical methods (HCA, PCA/FA) are often used in environmental research of pollution assessment of soils. These statistical methods play a main role in the effective evaluating of objects in the system and is categorized based on their similarities and properties [25,28,29,55,56,57]. Descriptive statistics and multivariate statistical methods (HCA, PCA) were performed using software STATISTICA^®^ software (version 12.5) and Microsoft Excel^®^ (2013).

## 3. Results and Discussion

### 3.1. The Total Concentration of Heavy Metals in Soils

The total concentrations of heavy metals in soils (Cd, Hg, Cu, Mn, Pb, V, Zn, Cr and Fe) from Ostrava and samples from district sites A, B and C, and the descriptive statistical analysis of heavy metals in the soil, are summarized in Table 1. The entering of heavy metals includes different emission sources of soil pollution by heavy metals, mainly traffic, industry emissions, local combustion of coal and municipal wastes. The extensive emissions of industrial dusts containing heavy metals originate from steel production processes and are the most important. The soils were divided in three classes (A: 1–10, B: 11–19 and C: 20–29) depending on pollution source distance (0–5 km, 5–10 km and 10–16 km). The total amount followed in order Hg ˂ Cd ˂ Fe ˂ Cr ˂ Cu ˂ Pb ˂ V ˂ Zn ˂ Mn.

The total metal concentration varied for Hg, 0.08–1.31 mg.kg^−1^ (median 0.19 ± 0.02), Pb, 11.09–174.03 mg.kg^−1^ (median 37.71 ± 3.04), Cd, 0.05–1.18 mg.kg^−1^ (median 0.21 ± 0.02), Cu, 4.88–98.83 mg.kg^−1^ (median 21.11 ± 1.63), Cr, 3.03–64.02 mg.kg^−1^ (median 17.46 ± 1.96), Fe, 4.45–20.64 mg.kg^−1^ (median 8.11 ± 0.58), Mn 263.72–2368.31 mg.kg^−1^ (median 1370.95 ± 71.53), V, 43.45–181.79 mg.kg^−1^ (median 96.72 ± 4.31) and Zn, 63.16–373.58 mg.kg^−1^ (median 204.57 ± 12.03).

The results of metal content from districts A, B and C confirmed that maximal highest concentration of Hg was in site A (1.31) > site C (0.25) > site B (0.19), Cd in site A (1.18) > site C (0.43) > site B (0.31), Fe in site A (20.64) ≈ site B (20.64) ≈ site C (20.49), Cr in site A (63.97) ≈ site B (63.54) ≈ site C (64.01), Cu in site C (98.83) ≈ site A (90.66) > site B (55.96); V in site A (154.48) > site A (181.79) ≈ site B (181.79), Pb in site A (174.24) > site C (162.02) > site A (110.03), Zn in site A (373.59) > site B (331.31) > site C (331.08) and Mn in site A (2368) > site C (2271) ≈ site B (2081). Generally, the highest concentration of Hg, Pb, Cd, Mn and Zn were identified in site A with a strong industry impact (steel production and metallurgical industry).

Moreover, the lowest metal concentration was found in the case of site B; however, the higher metal-contaminated soils of site C are due to the nearby chemical industry, as well as being an area with high traffic due to roads connecting to the D1 highway. The highest metal concentrations, mainly Hg (3, 5, 15 and 17 stations), Pb (4, 9, 10, 11, 21, 26 and 27 stations), Cd (9, 10, 11 stations), Cr (stations 3 and 15), Mn (23, 25 and 26 stations) and Zn (9, 10, 12 and 25 stations) were determined. The median of Hg, Pb and Cd concentrations exceeded background values and values of continental crust of metals in comparison with environmental background values and indicating that total area and sites A, B and C are being polluted considerably. According to comparison of maximum permissible limit values of potentially toxic metals (Pb, Cd, Cu, Cr, V and Zn), the median of metal concentrations was exceeded in the case of Pb in sites A and C as well as V and Mn in sites A, B and C. Nevertheless, the maximums of metal concentrations were exceeded in several location of sites A, B and C.

### 3.2. Pollution Assessment in Soils

On the basis of determined heavy metal concentrations in soil samples from Ostrava, the three single indices and five total complex indices (only integrated indices) were calculated. These indices indicated pollution by individual metals (I_geo_, EF and CF) and assessed soil quality (IPI, IPIT, PLI, C_deg_, mC_deg_ and PI_Nemerow_).

**Figure 3:**
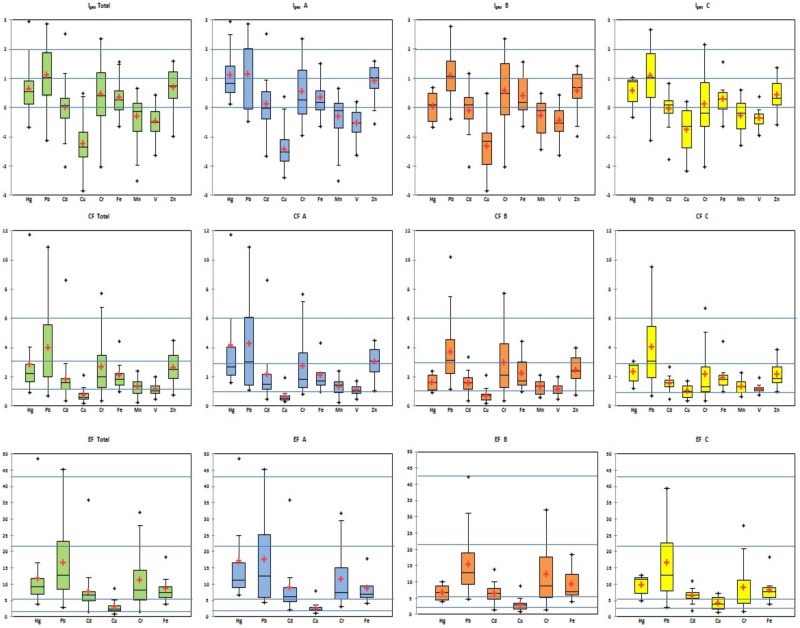
Box-and-whisker plot of single indices—geoaccumulation index (I_geo_), contamination factor (CF) and enrichment factor (EF).

The single indices—geoaccumulation index (I_geo_), enrichment factor (EF) and contamination factor (CF)—were used to assess the anthropogenic impact of heavy metal soil contamination. The metal concentrations of the geochemical background were used for calculation of the geoaccumulation index (I_geo_), and pre-industrial levels of metals were used for calculation of the contamination factor (CF). The concentration of Fe was used for calculation of enrichment factors (EF); this is characterized by relative stable concentrations in soils. Figure 3 represents the results of the single indices I_geo_, CF and EF of heavy metals in site A, B and C, as well as the total area of Ostrava, and in these graphs the classification levels of contamination are marked (blue line).

Box-and-whisker plots depict the mean, median, 1st and 3rd quartiles and extremes values (outliners) of single pollution indices calculated for soil samples in Ostrava (CZ). Boxes delineate the quartile range with an indication of the median (solid line); a small cross inside the box marks the mean.

The geoaccumulation index for Cd, Hg, Cu, Mn, Pb, V, Zn, Cr and Fe varied and values below zero indicate minimal anthropogenic effects with uncontaminated levels in the case of Cu, V and Mn in total area and also in sites A, B and C; nevertheless in case of Cd in site B and C. Values 0–1 determine that very low moderate contamination exists for Hg, Cr, Fe and Zn in total area and sites A, B and C, with an exception of Site A in case of Hg where I_geo_ is over 1. Moderate contaminations were verified in the case of Hg in Site A and Pb in all sites and total area of Ostrava where I_geo_ indices were over 1. Generally, the contents of heavy metals increase in the following order, Cu < V < Mn < Cd < Fe < Cr < Zn < Hg < Pb, based on I_geo_ average values. The minimal enrichment of metals in soils can be attributed to natural origin and fluctuations of metals if EF values are lower than 2. In case of copper obtained, EF values confirmed the moderate enrichment of Cu in soil samples. The mean and median values of EF values varied from 5 to 20 and values affirmed significant enrichment of Cd, Hg, Mn, Pb, V, Zn, Cr and Fe in soils in total area and sites A, B and C. EF values over 20 and 40 were obtained for Mn, Zn and V, and ones indicated enrichment levels from very high to extremely enriched. Based on the results, the contamination factors of Cu and V (CFs <  1) for sites A, B and C and total area of Ostrava can be regarded as localities with low contamination.

Box-and-whisker plot depicted the mean, median, 1st and 3rd quartiles and extremes values (outliners), and boxes delineate quartile range with indication of the median (solid line); a small cross inside the box marks the mean.

The CF levels from 1 to 3 with moderate contamination levels were obtained in occurrence of Cd, Hg, Mn, Pb, Zn, Cr and Fe with the exception of Pb. The CF levels growing follows Cu < V < Mn < Cd < Fe < Cr < Zn < Hg < Pb. The CF levels of Pb varied around 4, indicating considerable contamination of soils.

In this study, the integrated indices of pollution—integrated pollution index (IPI), pollution load index (PLI), Nemerow pollution index (PI_Nemerow_), degree of contamination (C_deg_) and modified contamination factor (mC_deg_)—were used for assessment of soil contamination by heavy metals.

**Figure 4:**
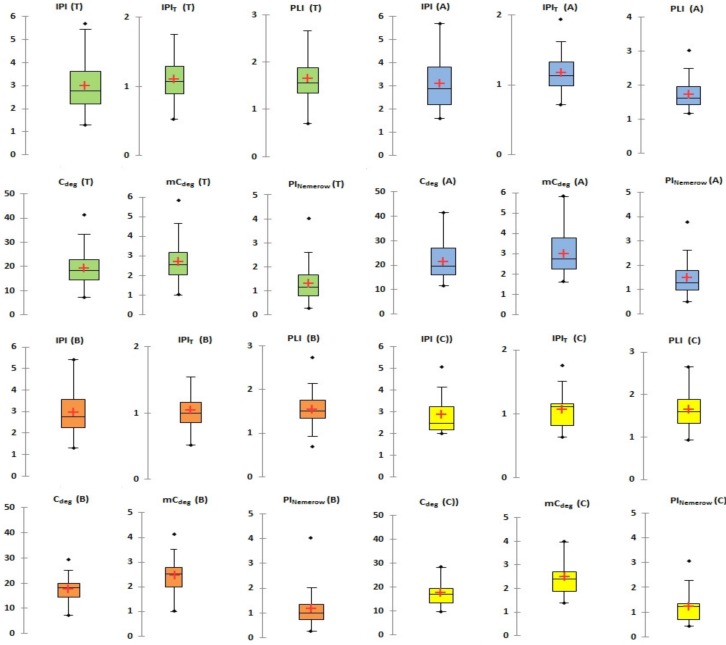
Box-and-whisker plot of integrated indices of pollution—integrated pollution index (IPI), integrated threshold pollution index (IPIT), pollution load index (PLI), degree of contamination (C_deg_), modified contamination factor (mC_deg_) and Nemerow pollution index (PI_Nemerow_).

The indices of pollution IPI confirmed strong pollution of the total area in Ostrava (median 2.761, mean 2.996) and also strong pollution of sites A (median 2.88, mean 3.091), B (median 2.77, mean 2.958) and C (median 2.48, mean 2.868). The mean of the threshold pollution index (IPIT) was in the case of total area 1.106, site A 1.177, site B 1.043 and site C 1.068, and the range of this index varied from 0.523 to 2.007, which means the level of pollution can be considered as moderate. The pollution load index (PLI) was over 1 (median 1.562 and mean 1.648) for total area and varied from 0.702 to 3.50, and thus the total area of Ostrava has to be considered as polluted through anthropogenic activity. The median of degree of contamination (C_deg_) was 18.23 for the total area of Ostrava where the median of this index also confirmed the considerable degree of contamination in case of site A (19.53), site B (18.10) and site C (17.10). The minimum of degree of contamination (C_deg_) was 7.255 (low degree of contamination) and the maximal value was 41.472 (very high degree of contamination); however, the median of C_deg_ was 18.288 for total area where the median of this index also confirmed the considerable degree of contamination in case of site A (19.53), site B (18.10) and site C (17.10). The calculated values over limit-values of pollution status (32) according to classifications, belong to soil samples from site A and from the sampling sites with closest proximity to industrialized sites (industrial metallurgical factory and long period active mines). Whereas the modified contamination factor (mC_deg_) for total area of Ostrava with median 2.560 classified this locality to class as moderately contaminated and individual sites A (2.73), B (2.53) and C (2.39) can be also included in the class moderately contaminated. Thus, the maximal value of mC_deg_ calculated 5.806 (A site) and the highest mC_deg_ were obtained in the case industrialized sites, and this value identifies a high contamination status for this area. The median of PI_Nemerow,_ with a value of 1.156 for total area and for sites A and C (1.29 and 1.23), indicate slight pollution and ranged from slight (nearly 1) to heavy pollution (4.028). Box-and-whisker plots of the integrated indices of pollution—IPI, IPIT, PLI, C_deg_, mC_deg_ and PI_Nemerow_, are illustrated in Figure 4.

### 3.3. Identification of Pollution Sources

#### 3.3.1. Correlation Analysis

The high correlation coefficients among heavy metals indicate the similar origin of pollution sources and very low or negative correlation coefficients reflect different sources, and it probably is closely related with natural or geogenic processes [9,43].

The Pearson’s correlation with statistical significance at *p* < 0.01 and *p* < 0.05 are summarized in Table 2. From a statistical viewpoint, the correlation coefficient (*r*) can be divided into four correlation classes: *r* ≤ 0.1 low, *r* in range 0.1–0.3 medium, *r* in range 0.3–0.5 high and *r* ≥ 0.5 mean very strong interrelationship among heavy metals. The high correlation among heavy metals could indicate that metals originated from a common pollution sources and could be characterized with a similar migration and transformation via physico-chemical conditions in the environment.

The high correlation among heavy metals observed in soils significantly reflect the anthropogenic input from industrial activities. Low or negative correlation of heavy metals in soils can also refer to others metal sources relating to natural processes [40,41,42,43].

The correlations in total area (*T*) were as follows: very strong correlation Fe–Cr (0.796), Pb–Cu (0.608) and high correlation among Pb–Cd (0.351), Pb–V (0.383), Cu–V (0.441), Mn–V (0.474) and V–Zn (0.407). Detailed analysis of the correlation matrix of sites A, B and C identified various pollution sources of heavy metals.

In Site A, very strong positive correlations were identified among Cr–Fe (0.676), Cr–Hg (0.526) and Cu–Pb (0.586), high correlation were among Fe–Hg (0.422), Pb–V (0.471) and V–Mn (0.368). In Site B very strong correlations were among Cr–Fe (0.901), Mn–Zn (0.741), V–Zn (0.720), Hg–Cd (0.551), Cd–Pb (0.587), Pb–Cu (0.605), Cd–Cu (0.645) and Cu–V (0.515). High correlations were over 0.3 in the case Hg–Pb (0.334), Hg–Cu (0.382), Pb–V (0.320), Cu–Zn (0.483) and Mn–V (0.495). In Site C very strong correlations were in the cases of Pb–Cu (0.893), Cr–Fe (0.828), Cr–Zn (0.787), Fe–Zn (0.727), Mn–V (0.699), Hg–Pb (0.513) and Hg–Cu (0.591). High correlation was confirmed for Pb–Cd (0.429), Pb–Mn (0.484), Pb–V (0.441), Cd–Cu (0.336), Cu–Mn (0.499), Cr–Mn (0.312 and Mn–Zn (0.412).

For Cr–Fe, Pb–Cu and Pb–V the correlations were strong and high in all cases (total area, sites A, B and C) and relationships decreased Cr–Fe (0.901—B, 0.893—C, 0.796—T, 0.676—A), Pb–Cu (0.893—C, 0.608—T, 0.605—B, 0.586—A) and Pb–V (0.471—A, 0.441—C, 0.383—T, 0.320—B).

The high and strong (*r* in the range from 0.3 to over 0.5) interrelationships were also for V–Mn and increased 0.368—A, 0.474—T, 0.495—B, 0.699—C. The strong and positive correlations indicate the conjoint main sources of heavy metals resulted in these areas from industry, combustion and traffic. From these dependencies obtained from the correlation analysis of total area, we can assume that the heavy metals as pollutants exist in three main groups Fe-Cr, Pb-Cu and Mn-V.

#### 3.3.2. Hierarchical Cluster Analysis (HCA)

**Figure 5:**
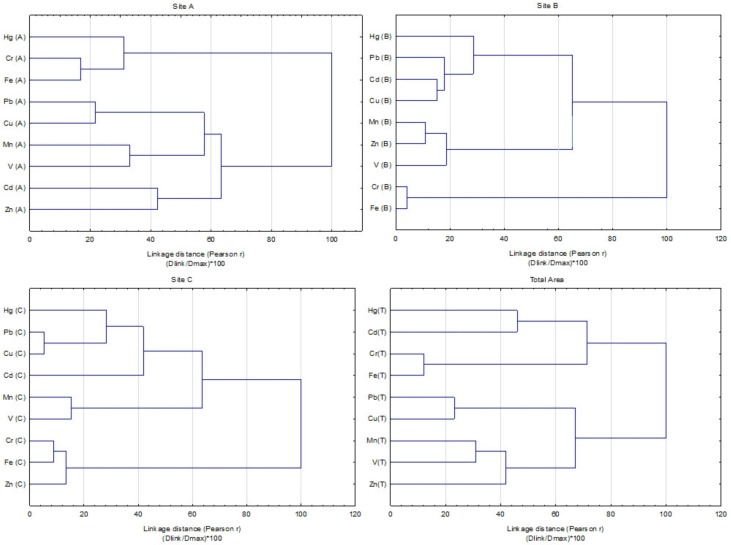
Dendrogram derived from hierarchical cluster analysis (HCA) of the heavy metal contents in the soils (Ward’s agglomeration method, Pearson *r* distance).

The dendrograms of hierarchical cluster analysis (HCA) of heavy metals concentration are presented in Figure 5. Hierarchical cluster analysis (HCA) identified several groups of association among metals in relation with area, and the metals associated in a group originated from the same pollution sources. In the case of the highly industrialized area, A, HCA identified four groups: Group I includes Fe–Cr and Hg joined this group, Pb and Cu formed Group II; Group III contain Mn and V, and IV Group consist metals Cd–Zn. The HCA analysis confirmed three groups of associated metals in area B, Group I are formed Fe–Cr, Group II include V–Mn and Zn, and Group III consist Cu, Pb, Cd and Hg. The three groups were identified also in area C; Group I—Cr and Fe with Zn, Group II Mn-V and group III Pb-Cu and Hg. Thereby the total area characterized four groups: Group I—Cr and Fe, Group II—Mn–V with Zn, group III—Pb–Cu and group IV—Cd–Hg. The correlation analysis CA (Table 2) also identified similar heavy metal interrelationships. The results from HCA analysis confirmed that grouped metals originated from the same pollution sources. The high content of Mn and Fe could be originated from weathering of parent materials. The presence of Fe and Mn could be indirect indicators of the Fe/Mn oxides in soils that influence heavy metals behavior in soils. Metals Pb, Cu and Cd, together with Hg resulted only from anthropogenic sources, such as industry, metallurgy, typical local combustion of low quality coal and different traffic intensity.

#### 3.3.3. Principal Component Analysis (PCA)/Factor Analysis (FA)

**Figure 6:**
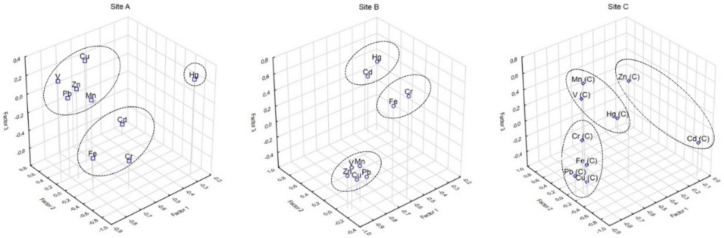
PCA/FA analysis results of Cd, Hg, Cu, Mn, Pb, V, Zn, Cr and Fe in soils of Ostrava (rotation: unrotated; extraction: principal components).

**Figure 7:**
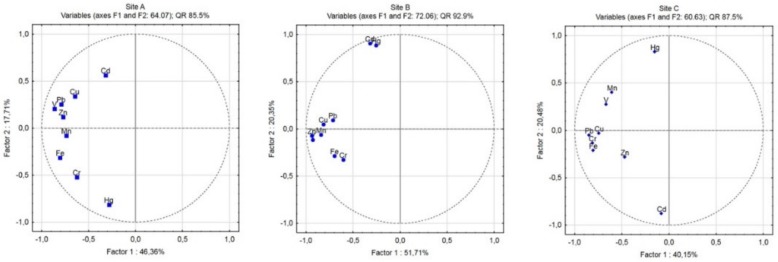
PCA loading plots of the first 2 principal components (F1, F2).

Multivariate statistical methods PCA/FA were performed to identify other sources and origins of the heavy metals in soils from three various sites of Ostrava (A, B and C). The Figure 6 and Figure 7 present the results obtained from PCA/FA.

Principal component analyses with factor analysis were applied for assessment and evaluation of the origin identification of metals in soils. The three principal components were extracted with eigenvalues over 1.0.

The results obtained from PCA/FA analysis for metals in soils showed that in the A site the first principal component (F1) described 46.36% of the total variance with high positive loadings V (0.922) Cu (0.801) and Zn ≅ Pb (0.765, 0.769); the second principal component (F2) explained 17.71% of total variance and included Cr (0.939) and Fe (0.862); and the third principal component (F3) consists of Cd (0.874) and explains 12.68% of the total variance. The heavy metals Cu and Zn (Pb) can be used for evaluation of long-term anthropogenic contamination mainly via stainless steel production and coal mine activities; high positive loadings of V can be attributed to the emissions from steel production of special steels, mainly high-strength steel. The other source of Cu and Pb is also attributed to the long-term high traffic situation in this area. The second principal component (F2) contains Fe (0.862) and Cr (0.939) and the main sources might be similar, such as distribution through fumes from industry (iron productions, blast furnace, roller factory, coke-oven plant, etc.). The third principal component (F3) consists from Cd (0.874) and explains 12.68% of the total variance and the main sources are extensive industry production.

In Site B, the FA/PCA results confirmed anthropogenic sources of metals. The first component consists of Cu (0.895), Zn (0.891), Pb (0.848), V (0.817) and Mn (0.784) and explains 51.71% of the total variance. The sources of these metals are the same as in Site A with the exception of Mn. The anthropogenic sources are the same as in Site A, where the main contributions are from enormous industry production. The second principal component explain 20.34% of total variance and includes Hg (0.973) and Cd (0.960) and suggests the main contributions of these metals might be attributed to local coal combustions. The third component accounted for 15.31% of the total variance and contributed to Cr (0.953) and Fe (0.932), and these metals indicated a similar sources (metallurgical industry).

In the Site C, the results obtained from FA/PCA analysis were slightly different. The first principal component explains 40.14% of total variance with high values for Cu (0.905), Pb (0.902), Fe (0.794) and Cr (0.621). The metal Cu and Pb indicated long-term pollution from two main sources, steelworks industry and high vehicle traffic density. The Fe and Cr released from metallurgical activities in this region, and its distribution, is also on long distance. The second principal component with 20.47% of the total variance belongs to Cd (0.814); the main contributions of this metals include the high level of fossil fuel, local combustions and also the applications of manure and agrochemicals in this peripheral and almost rural site in this region. The third principal component includes Mn (0.813), Zn (0.743) and V (0.737) that accounts for 16.19% of the total variance.

Due to the persistent and accumulation properties of heavy metals in soils, they can be considered as markers of long-term anthropogenic pollution. The common group of metals in Site A, B and C included Fe–Cr with association of Cd in Site A and Pb–Cu in Site C. These major groups originated mainly from coal consumption (industry, metallurgy and local combustion of low-quality coal). The household local combustion of coal can be considered as potentially problematic with serious environmental and health impacts in the Ostrava region due to different fuel utilization and also with pollution emission control. The presence of Pb in the Ostrava region originates from the burning of coal in industries and households and metallurgy, as well as industrial wastes [10,11,12,13,16,24]. The Cu derived mainly from metal industries, steel production and also traffic.

### 3.4. Ecological Risk Assessment

**Figure 8:**
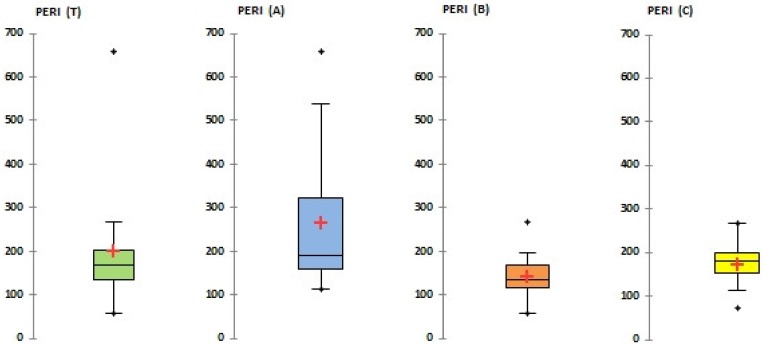
Box-and-whisker plot of the potential ecological risk index (PERI).

The values of potential ecological risk (PERI) in the studied soil samples varied (see Figure 8). The PERI values of Site A with high industrial affected soils ranged from 112 to 654 with a median of 190, which means a strong ecological risk, whereas approximately 45% of PERI values lied between 90 and 180 and indicated a moderate ecological risk; 31% were between 180 and 360 that indicated a strong ecological risk; 21% were between 360 and 600 and indicated a very strong ecological risk; and 3% were over 600 and indicated a highly strong ecological risk. The levels of PERI in Site B varied from 59 to 195 with a median of 125; this means that the area can be characterized as with a moderate ecological risk, whereas approximately 21% of PERI values lied below 90, indicating low ecological risk; 73% were between 90 and 180 that indicated a moderate ecological risk; and 6% were between 180 and 360 and indicated a strong ecological risk. The PERI values from soil samples from the suburban C site ranged from 73 to 266 with a median of 172 that can be marked as an area with moderate risk; approximately 55% were between 90 and 180 and 45% were between 180 and 360.

The total study area (T) can be assigned as with a moderate ecological risk (PERI 168), but approximately 46% of all PERI values lied between 90 and 180 and indicated a moderate ecological risk; 41% were between 180 and 360 that indicated a strong ecological risk; 10% were between 360 and 600 and indicated a very strong ecological risk; and 3% were over 600 and indicated a highly strong ecological risk.

Box-and-whisker plots depicted mean, median, 1st and 3rd quartiles and extremes values (outliners); boxes delineate quartile range with indication of the median (solid line); and a small cross inside the box marks the mean.

### 3.5. Health Risk Assessment

The human health risk assessment was evaluated using non-carcinogenic hazards by calculation of the hazard index (*HI*) and carcinogenic risk over lifetime (*LCR*) for adult and child (Equations (1)–(6)). The individual organism is exposed via three pathways (oral ingestion, inhalation and dermal contact) and these exposures are calculated as chronic daily intake (Table S3, Supplementary Materials) with RfD (Table S4, Supplementary Materials).

Hazard index (*HI*) indicates the cumulative non-carcinogenic risk and the highest values of *HI* were in the case Pb in Site C for adults and all areas for children; the cumulative hazard index was estimated for all areas for V for children. In these cases, the *HIs* were over 1 and adverse health effects are possible.

The mean *HI* values of children’s non carcinogenic risk for total area decrease in the following order: V > Pb > Zn > Cr > Mn > Hg > Cu > Cd > Fe.

The non-carcinogenic risk of exposure of heavy metals from soils for children is higher due to different physiological properties in contrast to adults. From Table 3 it can be seen that *HI* values for adults and children (Table 3) are different for the values for individual metals as well as for the studied areas. For children, the riskiest site is A.

The carcinogenic risk of Pb, Cd and Cr via non-dietary exposure in soils were calculated and its sum expressed total cancer risk over a lifetime (*LCR*) (Table 4). The range of tolerable values (1.0 × 10^−6^ to 1.0 × 10^−4^) was obtained in the case of Pb, Cd and Cr for the adult across the whole study area and the acceptable threshold limit for adults were 1.20 × 10^−4^ for Cr.

The range of carcinogenic risk levels could be characterized in detail based on the level range: the very low (<10^−6^), low (10^−6^–10^−5^), medium (10^−5^–10^−4^), high (10^−4^–10^−3^), and very high (>−10^−3^) [58,59,60]. For adults, the low carcinogenic risk is for Pb, medium is for Cd and high is for Cr.

There is a medium carcinogenic risk for a child in the case of Pb, but with a high risk for Cd and very high for Cr. Generally, it is assumed that the level of Cd and Cr for children is not acceptable and exceeded the acceptable threshold values in whole area. The exceeded values of 10^−4^ in the case Cd and Cr for a child and in the case of Cr for adult expresses a carcinogenic risk to humans.

## 4. Conclusions

This study presents a detailed study of heavy metal pollution in soils of Ostrava with regard to type of site (industrial, urban or suburban with traffic load). The main influence on the metal distribution and its accumulation in soil were due to the type of land used (industrial, urban) and transport and represent a high risk for soil and environment pollution, as well as for human health. According the heavy metal assessment in soil from Ostrava and the interpretation of the obtained results, it can be stated that

■ on the basis of I_geo_ and CF values, the levels of heavy metals risen in the following order Cu < V < Mn < Cd < Fe < Cr < Zn < Hg < Pb;
■ indices of pollution confirmed anthropogenic pollution of the total area in Ostrava from strong to moderate levels;
■ results from correlation analysis confirmed the assumption that heavy metals in soils create three major groups (Fe–Cr, Pb–Cu and Mn–V), and hierarchical cluster analysis identified several groups among heavy metals in relation to area and common pollution source of heavy metals, whereas principal component analyses with factor analysis were applied for pollution assessment and identification of the origin source of heavy metals;
■ values of potential ecological risk index varied, and its value indicate a moderate ecological risk, whereas several sites with very strong ecological risk and highly strong ecological risk exist in Ostrava; and
■ a serious health carcinogenic risk was identified in the case of Pb, and the high risk is for Cd and very high for Cr for children.

## Figures and Tables

**Table 1:** Descriptive statistics of total concentration of Cd, Hg, Cu, Mn, Pb, V, Zn, Cr and Fe (mg.kg^−1^) in soil samples of Ostrava.

	Statistic	Hg	Pb	Cd	Cu	Cr	Fe	Mn	V	Zn
**Total**	Min	0.08	11.09	0.05	4.88	3.03	4.45	263.72	43.45	60.91
	Max	1.31	174.03	1.18	98.83	64.02	20.64	2368.31	181.79	393.08
	Median	0.19	37.71	0.21	21.11	17.46	8.11	1370.92	96.71	204.569
	SD	0.02	3.04	0.02	1.63	1.96	0.58	71.53	4.31	12.03
**Site A**	Min	0.13	17.03	0.06	13.88	3.379	4.502	263.722	43.455	85.160
	Max	1.31	174.24	1.18	90.66	63.974	20.644	2368.786	154.488	373.587
	Median	0.34	56.56	0.265	27.28	19.75	9.76	1327.51	97.79	250.03
	SD	0.05	3.33	0.05	1.09	2.793	0.839	113.230	5.054	15.707
**Site B**	Min	0.08	18.22	0.05	4.88	3.026	4.455	557.329	43.455	63.160
	Max	0.19	110.03	0.31	55.96	63.539	20.644	2081.199	181.798	331.306
	Median	0.12	42.47	0.19	31.50	17.058	7.811	1370.924	90.593	195.704
	SD	0.01	5.94	0.01	2.95	3.965	1.177	111.235	7.988	17.912
**Site C**	Min	0.09	11.09	0.06	15.01	3.03	4.455	557.329	69.291	78.906
	Max	0.25	162.02	0.43	98.83	64.015	20.493	2271.312	181.798	331.081
	Median	0.19	55.61	0.21	37.78	16.618	8.473	1325.369	104.784	167.727
	SD	0.05	46.71	0.08	24.01	16.107	4.828	510.931	30.657	92.971
**Earth crust**	a	0.08	16.00	0.13	47.00	83.00	46500	1000.00	90.00	83.000
	b	0.05	17	0.09	28	92.0	-	-	97	67
	c	0.07	15	0.1	55	100	-	900	135	70
**PEL, CZ**		0.60	50	0.40	50	40	-	-	50	100
**B_n_**		0.12	14.58	0.12	8.35	3.03	5.46	263.0	4.053	61.87

-: no relevant data; Min: minimum, Max: maximum, SD: standard deviation; a [36], b [35], c [37]; PEL—permissible limit—Decree No. 153/2016 Coll. in CZ; *B_n_*—concentration of heavy metal (*n*) geochemical background.

**Table 2:** Correlation matrix (Pearson) of total metal contents in soil samples from Ostrava.

	Hg (T)	Pb (T)	Cd (T)	Cu (T)	Cr (T)	Fe (T)	Mn (T)	V (T)	Zn (T)
**Hg (T)**	1.000	0.004	0.222	−0.097	0.291 *	0.245 *	0.118	−0.172	0.190
**Pb (T)**		1.000	0.351 **	0.608 **	−0.050	0.089	0.197	0.383 **	0.174
**Cd (T)**			1.000	0.111	−0.042	0.105	−0.044	0.035	0.236
**Cu (T)**				1.000	−0.149	−0.025	0.120	0.411 **	0.054
**Cr (T)**					1.000	0.796 **	0.066	0.085	0.211
**Fe (T)**						1.000	0.138	0.159	0.128
**Mn (T)**							1.000	0.474 *	0.268 **
**V (T)**								1.000	0.407 *
**Zn (T)**									1.000
	**Hg (A)**	**Pb (A)**	**Cd (A)**	**Cu (A)**	**Cr (A)**	**Fe (A)**	**Mn (A)**	**V (A)**	**Zn (A)**
**Hg (A)**	1.000	−0.143	0.126	−0.193	0.526 **	0.422 *	0.156	−0.269	0.043
**Pb (A)**		1.000	0.326	0.586 **	0.009	0.313	0.108	0.471 **	0.067
**Cd (A)**			1.000	0.057	0.036	0.281	−0.110	0.027	0.194
**Cu (A)**				1.000	−0.220	0.005	−0.187	0.360	−0.016
**Cr (A)**					1.000	0.676 **	0.059	−0.239	0.088
**Fe (A)**						1.000	0.259	0.065	0.035
**Mn (A)**							1.000	0.368 *	−0.102
**V (A)**								1.000	0.361
**Zn (A)**									1.000
	**Hg (B)**	**Pb (B)**	**Cd (B)**	**Cu (B)**	**Cr (B)**	**Fe (B)**	**Mn (B)**	**V (B)**	**Zn (B)**
**Hg (B)**	1.000	0.334	0.551 **	0.382	0.072	0.037	0.212	0.235	0.180
**Pb (B)**		1.000	0.587 **	0.605 **	−0.204	−0.244	0.139	0.320	0.282
**Cd (B)**			1.000	0.645 **	−0.399	−0.319	0.175	0.164	0.230
**Cu (B)**				1.000	−0.028	0.016	0.196	0.515 **	0.438 *
**Cr (B)**					1.000	0.901 **	−0.040	0.286	0.090
**Fe (B)**						1.000	−0.070	0.221	0.021
**Mn (B)**							1.000	0.495 *	0.741 **
**V (B)**								1.000	0.720 **
**Zn (B)**									1.000
	**Hg (C)**	**Pb (C)**	**Cd (C)**	**Cu (C)**	**Cr (C)**	**Fe (C)**	**Mn (C)**	**V (C)**	**Zn (C)**
**Hg (C)**	1.000	0.513	0.240	0.591 *	0.162	0.239	0.261	−0.037	0.277
**Pb (C)**		1.000	0.429	0.893 **	0.099	0.141	0.484	0.441	0.219
**Cd (C)**			1.000	0.336	0.121	-0.101	−0.055	0.252	0.169
**Cu (C)**				1.000	−0.102	−0.039	0.499	0.265	0.125
**Cr (C)**					1.000	0.828 **	0.312	0.300	0.787 **
**Fe (C)**						1.000	0.291	0.271	0.727 **
**Mn (C)**							1.000	0.699 **	0.412
**V (C)**								1.000	0.270
**Zn (C)**									1.000

* Coefficients correlations are significant at *p* < 0.05 (2-tailed); ** Coefficients correlations are significant at *p* < 0.01(2-tailed).

**Table 3:** Cumulative hazard index (HI) for non-carcinogenic risk.

		Hg	Pb	Cd	Cu	Cr	Fe	Mn	V	Zn
**Adult**	Total	8.71.10^−3^	1.48.10^−1^	9.63.10^−4^	7.83.10^−3^	8.00.10^−2^	1.59.10^−4^	3.77.10^−2^	1.90.10^−1^	9.38.10^−2^
	Site A	1.56.10^−2^	2.22.10^−1^	1.21.10^−3^	1.01.10^−2^	9.05.10^−2^	1.92.10^−4^	3.65.10^−2^	1.92.10^−1^	1.15.10^−1^
	Site B	5.50.10^−3^	1.67.10^−1^	8.71.10^−4^	1.17.10^−2^	7.82.10^−2^	1.53.10^−4^	3.77.10^−2^	1.78.10^−1^	8.97.10^−2^
	Site C	8.71.10^−3^	2.19.10^−0^	9.63.10^−4^	1.40.10^−2^	7.62.10^−2^	1.66.10^−4^	3.65.10^−2^	2.06.10^−1^	7.69.10^−2^
**Child**	Total	8.11.10^−2^	1.38.10^−0^	8.97.10^−3^	7.29.10^−2^	7.46.10^−1^	1.48.10^−3^	3.51.10^−1^	1.77.10^−0^	8.74.10^−1^
	Site A	1.45.10^−1^	2.07.10^−0^	1.13.10^−2^	9.42.10^−2^	8.43.10^−1^	1.79.10^−3^	3.40.10^−1^	1.79.10^−0^	1.07.10^−0^
	Site B	5.12.10^−2^	1.55.10^−0^	8.11.10^−3^	1.09.10^−2^	7.28.10^−1^	1.43.10^−3^	3.51.10^−1^	1.66.10^−0^	8.36.10^−1^
	Site C	8.11.10^−2^	2.04.10^−0^	8.97.10^−3^	1.30.10^−2^	7.10.10^−1^	1.55.10^−3^	3.40.10^−1^	1.92.10^−0^	7.16.10^−1^

**Table 4:** Total carcinogenic risk over lifetime (LCR) for adult and child.

		Pb	Cd	Cr
**Adults**	Total	4.41.10^−6^	1.82.10^−5^	1.20.10^−4^
	Site A	6.61.10^−6^	2.30.10^−5^	1.36.10^−4^
	Site B	4.96.10^−6^	1.65.10^−5^	1.17.10^−4^
	Site C	6.50.10^−6^	1.82.10^−5^	1.14.10^−4^
**Child**	Total	4.11.10^−5^	1.69.10^−4^	1.12.10^−3^
	Site A	6.16.10^−5^	2.14.10^−4^	1.27.10^−3^
	Site B	4.62.10^−5^	1.53.10^−4^	1.09.10^−3^
	Site C	6.06.10^−5^	1.69.10^−4^	1.06.10^−3^

## Supplementary Materials

The following are available online at https://www.mdpi.com/1660-4601/16/22/4495/s1, Table S1: Matrix composition of METRANAL 34 - loam soil with higher contents of elements. Table S2: Formula and limit value of pollution indices calculated in this study. Table S3: Chronic daily intake (*CDI*) of metals. Table S4: *RfD* of different metals for different exposure pathway [49,50,51,52,59,60].
