# Supplementary material for: Potential Ecological Risk and Human Health Risk Assessment of Heavy Metal Pollution in Industrial Affected Soils by Coal Mining and Metallurgy in Ostrava, Czech Republic
Source: Int J Environ Res Public Health. 2019 Nov 14;16(22):4495. doi: 10.3390/ijerph16224495 (PMC6888271; doi:10.3390/ijerph16224495)
Supplement: Supplementary file 1 [file ijerph-16-04495-s001.pdf]

## Supplementary materials

**Table S1.** Matrix composition of METRANAL 34 - loam soil with higher contents of elements.

| **Oxides** | **Matrix composition (wt. % of the oxides - dry weight)** |
|---|---|
| $SiO_2$ | 64.35 |
| $Al_2O_3$ | 13.10 |
| CaO | 2.07 |
| MgO | 1.29 |
| $Fe_2O_3$ | 5.82 |
| $K_2O$ | 2.55 |
| $Na_2O$ | 0.72 |
| $P_2O_5$ | 0.45 |
| $TiO_2$ | 1.32 |
| loss on ignition at 900°C | 7.87 |

**Table S2.** Formula and limit value of pollution indices calculated in this study.

| | Indices and formula | Limit value |
|---|---|---|
| **Single indices** | Geoaccumulation index ($I_{geo}$) $I_{geo} = log_2(\frac{C_n}{1.5 B_n})$ | ≤ 0 Unpolluted, 0 – 1 Unpolluted to moderately polluted, 1 – 2 Moderately polluted, 2 – 3 Moderately to highly polluted, 3 – 4 Highly polluted, 4 – 5 Highly to extremely highly polluted, ≥ 5 Extremely highly polluted, |
| | Enrichment factor (EF) $EF_i = \frac{\frac{C_{n(sample)}}{C_{Ref\ (sample)}}}{\frac{B_{n(background)}}{B_{Ref\ (background)}}}$ | ≤ 2 Minimal enrichment, 2 – 5 Moderate enrichment, 5 – 20 Significant enrichment, 20 – 40 Very high enrichment, ≥ 40 Extremely high enrichment |
| | Contamination factor (CF) $CF_i = \frac{C_M}{C_{np}}$ | ≤ 1 Low contamination, 1 – 3 Moderate contamination, 3 – 6 Considerable contamination, ≥ 6 Very high contamination |
| | Single pollution index (PI) $PI_i = \frac{C_n}{B_n}$ | ≤ 1 non-pollution, 1 – 2 low level of pollution , 2 – 3 moderate level of pollution, 3 – 5 strong level of pollution, ≥ 5 very strong level of pollution |
| **Total complex indices** | Pollution load index (PLI) $PLI = \sqrt[n]{PI_1 . PI_2 . PI_3 ... PI_n}$ | < 1 No polluted, 1 Baseline of pollution, >1 Polluted |
| | Integrated threshold pollution index ($IPI_T$) $IPI_T = \frac{1}{n} \sum_{i=1}^{n} \frac{C_n}{C_{TL}}$ | ≤ 1 low polluted, 1 – 3 Moderate polluted, ≥ 3 Strong polluted |
| | Degree of contamination ($C_{deg}$) $C_{deg} = \sum_{i=1}^{n} CF_i$ | ≤ 8 Low degree of contamination, 8 – 16 Moderate degree of contamination, 16 – 32 Considerable degree of contamination, ≥ $C_{deg}$ Very high degree of contamination |
| | Modified contamination factor ($mC_{deg}$) $mC_{deg} = \frac{1}{n} \sum_{i=1}^{n} CF_i$ | ≤ 1.5 Very low contamination, 1.5 – 2 Low contamination, 2 – 4 Moderate contamination 4 – 8 High contamination, 8 – 16 Very high contamination, 16 – 32 Extremely high contamination, ≥ 32 Ultra high contamination |
| | Nemerow Pollution Index ($PI_{Nemerow}$) $PI_{Nemerow} = \sqrt{\frac{\left(\frac{1}{n} \sum_{i=1}^{n} PI_i\right)^2 + P_{iMax}^2}{2}}$ | ≤0.7 Clean, 0.7 – 1 Warning limit, 1–2 Slight pollution, 2–3 Moderate pollution, ≥3Heavy pollution |
| | Risk index (PERI) $PERI = \sum_{i=1}^{n} E_r^i\ ;\ E_r^i = T_r^i . C_f^i$ | ≤ 90 Low, 90 – 180 Moderate, 180 – 360 Strong, 360 – 720 Very strong, ≥ 720 Highly strong |

*Explanation*: $C_n$ - heavy metal content in soil, $B_n$ - concentration of heavy metal (*n*) geochemical background (factor 1.5 - compensating the $B_n$ due to to to lithogenic effects, $C_n$ *(sample)* - metal concentration in soil analyzed sample, $C_{Ref}$*(sample)* - concentration of the reference metal in soil analyzed sample, $B_{n(background)}$ - metal concentration in the reference environment, $B_{Ref}$*(background)* - reference metal concentration in the reference environment, $C_{min}$ - minimum concentration, $C_{max}$ - maximum concentration, $C_{TL}$ - tolerance levels of metal concentration, $C_M$ - mean metal concentration, $C_{np}$ - preindustrial concentration of metal, *i, i, m* - number of determined heavy metals, $E_r^i$ potential risk of individual metal, $T_r^i$ toxic response factor

**Table S3.** Chronic daily intake (*CDI)* of metals.

| | area | Hg | Pb | Cd | Cu | Cr | Fe | Mn | V | Zn |
|---|---|---|---|---|---|---|---|---|---|---|
| $CDI_{Ing}(a)$ | Total | $2.60.10^{-6}$ | $5.17.10^{-4}$ | $2.88.10^{-6}$ | $2.89.10^{-4}$ | $2.39.10^{-4}$ | $1.11.10^{-4}$ | $1.88.10^{-2}$ | $1.32.10^{-3}$ | $2.80.10^{-3}$ |
| | Site A | $4.66.10^{-6}$ | $7.75.10^{-4}$ | $3.63.10^{-6}$ | $3.74.10^{-4}$ | $2.71.10^{-4}$ | $1.34.10^{-4}$ | $1.82.10^{-2}$ | $1.34.10^{-3}$ | $3.43.10^{-3}$ |
| | Site B | $1.64.10^{-6}$ | $5.82.10^{-4}$ | $2.60.10^{-6}$ | $4.32.10^{-4}$ | $2.34.10^{-4}$ | $1.07.10^{-4}$ | $1.88.10^{-2}$ | $1.24.10^{-3}$ | $2.68.10^{-6}$ |
| | Site C | $2.60.10^{-6}$ | $7.62.10^{-4}$ | $2.88.10^{-6}$ | $5.18.10^{-4}$ | $2.28.10^{-4}$ | $1.16.10^{-4}$ | $1.82.10^{-2}$ | $1.44.10^{-3}$ | $2.30.10^{-3}$ |
| $CDI_{Derm}(a)$ | Total | $1.04.10^{-8}$ | $2.06.10^{-6}$ | $1.15.10^{-8}$ | $1.15.10^{-6}$ | $9.54.10^{-7}$ | $4.43.10^{-7}$ | $7.49.10^{-5}$ | $5.29.10^{-6}$ | $1.12.10^{-5}$ |
| | Site A | $1.86.10^{-8}$ | $3.09.10^{-6}$ | $1.45.10^{-8}$ | $1.49.10^{-6}$ | $1.08.10^{-6}$ | $5.33.10^{-7}$ | $7.26.10^{-5}$ | $5.34.10^{-6}$ | $1.37.10^{-5}$ |
| | Site B | $6.56.10^{-9}$ | $2.32.10^{-6}$ | $1.04.10^{-8}$ | $1.72.10^{-6}$ | $9.32.10^{-7}$ | $4.27.10^{-7}$ | $7.49.10^{-5}$ | $4.95.10^{-6}$ | $1.07.10^{-5}$ |
| | Site C | $1.04.10^{-8}$ | $3.04.10^{-6}$ | $1.15.10^{-8}$ | $2.06.10^{-6}$ | $9.08.10^{-7}$ | $4.63.10^{-7}$ | $7.24.10^{-5}$ | $5.73.10^{-6}$ | $9.17.10^{-6}$ |
| $CDI_{Inh}(a)$ | Total | $4.59.10^{-11}$ | $9.12.10^{-9}$ | $5.08.10^{-11}$ | $5.10.10^{-9}$ | $4.22.10^{-9}$ | $1.96.10^{-9}$ | $3.31.10^{-7}$ | $2.34.10^{-8}$ | $4.95.10^{-8}$ |
| | Site A | $8.22.10^{-11}$ | $1.37.10^{-8}$ | $6.41.10^{-11}$ | $6.59.10^{-9}$ | $4.77.10^{-9}$ | $2.36.10^{-9}$ | $3.21.10^{-7}$ | $2.36.10^{-8}$ | $6.04.10^{-8}$ |
| | Site B | $2.90.10^{-11}$ | $1.03.10^{-8}$ | $4.59.10^{-11}$ | $7.61.10^{-9}$ | $4.12.10^{-9}$ | $1.89.10^{-9}$ | $3.31.10^{-7}$ | $2.19.10^{-8}$ | $4.73.10^{-8}$ |
| | Site C | $4.59.10^{-11}$ | $1.34.10^{-8}$ | 5.08E-11 | $9.13.10^{-6}$ | $4.02.10^{-9}$ | $2.05.10^{-9}$ | $3.20.10^{-7}$ | $2.53.10^{-8}$ | $4.05.10^{-8}$ |
| $CDI_{Ing}(b)$ | Total | $2.43.10^{-5}$ | $4.82.10^{-3}$ | $2.68.10^{-5}$ | $2.70.10^{-3}$ | $2.23.10^{-3}$ | $1.04.10^{-3}$ | $1.75.10^{-1}$ | $1.24.10^{-2}$ | $2.62.10^{-2}$ |
| | Site A | $4.35.10^{-5}$ | $7.23.10^{-3}$ | $3.39.10^{-5}$ | $3.49.10^{-3}$ | $2.53.10^{-3}$ | $1.25.10^{-3}$ | $1.70.10^{-1}$ | $1.25.10^{-2}$ | $3.20.10^{-2}$ |
| | Site B | $1.53.10^{-5}$ | $5.43.10^{-3}$ | $2.43.10^{-5}$ | $4.03.10^{-3}$ | $2.18.10^{-3}$ | $9.99.10^{-4}$ | $1.75.10^{-1}$ | $1.16.10^{-2}$ | $2.50.10^{-2}$ |
| | Site C | $2.43.10^{-5}$ | $7.11.10^{-3}$ | $2.68.10^{-5}$ | $4.83.10^{-3}$ | $2.12.10^{-3}$ | $1.08.10^{-3}$ | $1.69.10^{-1}$ | $1.34.10^{-2}$ | $2.14.10^{-2}$ |
| $CDI_{Derm}(b)$ | Total | $4.85.10^{-8}$ | $9.62.10^{-6}$ | $5.36.10^{-8}$ | $5.38.10^{-6}$ | $4.45.10^{-6}$ | $2.07.10^{-6}$ | $3.50.10^{-4}$ | $2.47.10^{-5}$ | $5.22.10^{-5}$ |
| | Site A | $8.67.10^{-8}$ | $1.44.10^{-5}$ | $6.76.10^{-8}$ | $6.96.10^{-6}$ | $5.04.10^{-6}$ | $2.49.10^{-6}$ | $3.39.10^{-4}$ | $2.49.10^{-5}$ | $6.38.10^{-5}$ |
| | Site B | $3.06.10^{-8}$ | $1.08.10^{-5}$ | $4.85.10^{-8}$ | $8.03.10^{-6}$ | $4.35.10^{-6}$ | $1.99.10^{-6}$ | $3.50.10^{-4}$ | $2.31.10^{-5}$ | $4.99.10^{-5}$ |
| | Site C | $4.85.10^{-8}$ | $1.42.10^{-5}$ | $5.36.10^{-8}$ | $9.64.10^{-6}$ | $4.24.10^{-6}$ | $2.16.10^{-6}$ | $3.38.10^{-4}$ | $2.67.10^{-5}$ | $4.28.10^{-5}$ |
| $CDI_{Inh}(b)$ | Total | $2.14.10^{-10}$ | $4.25.10^{-8}$ | $2.37.10^{-10}$ | $2.38.10^{-8}$ | $1.97.10^{-8}$ | $9.15.10^{-9}$ | $1.55.10^{-6}$ | $1.09.10^{-7}$ | $2.31.10^{-7}$ |
| | Site A | $3.84.10^{-10}$ | $6.38.10^{-8}$ | $2.99.10^{-10}$ | $3.08.10^{-8}$ | $2.23.10^{-8}$ | $1.10.10^{-8}$ | $1.50.10^{-6}$ | $1.10.10^{-7}$ | $2.82.10^{-7}$ |
| | Site B | $1.35.10^{-10}$ | $4.79.10^{-8}$ | $2.14.10^{-10}$ | $3.55.10^{-8}$ | $1.92.10^{-8}$ | $8.81.10^{-9}$ | $1.55.10^{-6}$ | $1.02.10^{-7}$ | $2.21.10^{-7}$ |
| | Site C | $2.14.10^{-10}$ | $6.27.10^{-8}$ | $2.37.10^{-10}$ | $4.26.10^{-8}$ | $1.87.10^{-8}$ | $9.56.10^{-9}$ | $1.50.10^{-6}$ | $1.18.10^{-7}$ | $1.89.10^{-7}$ |

$CDI_x(a)$ - Chronic daily intake (CDI) of metals for adults

$CDI_x(b)$ - Chronic daily intake (CDI) of metals for child

**Table S4.** *RfD* of different metals for different exposure pathway [49-52,59-60].

| | Hg | Pb | Cd | Cu | Cr | Fe | Mn | V | Zn |
|---|---|---|---|---|---|---|---|---|---|
| $RfD_{Ing}$ | $3.00.10^{-4}$ | $3.50.10^{-3}$ | $1.00.10^{-3}$ | $4.00.10^{-2}$ | $3.00.10^{-3}$ | $3.00.10^{-3}$ | $4.60.10^{-2}$ | $7.00.10^{-3}$ | $3.00.10^{-1}$ |
| $RfD_{Derm}$ | $1.23.10^{-4}$ | $5.25.10^{-4}$ | $1.00.10^{-5}$ | $1.2.10^{-2}$ | $6.10^{-5}$ | $4.50.10^{-2}$ | $1.84.10^{-3}$ | $7.00.10^{-5}$ | $6.00.10^{-2}$ |
| $RfD_{Inh}$ | $3.01.10^{-4}$ | $3.45.10^{-3}$ | $5.70.10^{-5}$ | $4.00.10^{-2}$ | $2.86.10^{-5}$ | $3.00.10^{-3}$ | $1.43.10^{-5}$ | $7.00.10^{-3}$ | $3.00.10^{-1}$ |
